# High intensity interval training exercise-induced physiological changes and their potential influence on metabolic syndrome clinical biomarkers: a meta-analysis

**DOI:** 10.1186/s12902-020-00640-2

**Published:** 2020-11-10

**Authors:** I. Serrablo-Torrejon, A. Lopez-Valenciano, M. Ayuso, E. Horton, X. Mayo, G. Medina-Gomez, G. Liguori, A. Jimenez

**Affiliations:** 1 Faculty Research Centre for Sport, Exercise & Life Sciences, School of Health and Life Sciences, Coventry University, Coventry, UK; 2 GO fit LAB, Av. Islas de Filipinas, 7, 28003 Madrid, Spain; 3 Observatory of Healthy & Active Living, Spain Active Foundation, Centre for Sport Studies, King Juan Carlos University, Madrid, Spain; 4 University of Rhode Island, Kingston, RI USA; 5 Advanced Wellbeing Research Centre, College of Health, Wellbeing and Life Sciences, Sheffield Hallam University, Sheffield, UK

**Keywords:** High intensity interval training, Metabolic syndrome, Meta-analysis

## Abstract

**Background:**

Despite the current debate about the effects of high intensity interval training (HIIT), HIIT elicits big morpho-physiological benefit on Metabolic Syndrome (MetS) treatment. However, no review or meta-analysis has compared the effects of HIIT to non-exercising controls in MetS variables. The aim of this study was to determine through a systematic review, the effectiveness of HIIT on MetS clinical variables in adults.

**Methods:**

Studies had to be randomised controlled trials, lasting at least 3 weeks, and compare the effects of HIIT on at least one of the MetS clinical variables [fasting blood glucose (BG), high-density lipoprotein (HDL-C) triglyceride (TG), systolic (SBP) or diastolic blood pressure (DBP) and waist circumference (WC)] compared to a control group. The methodological quality of the studies selected was evaluated using the PEDro scale.

**Results:**

Ten articles fulfilled the selection criteria, with a mean quality score on the PEDro scale of 6.7. Compared with controls, HIIT groups showed significant and relevant reductions in BG (− 0.11 mmol/L), SBP (− 4.44 mmHg), DBP (− 3.60 mmHg), and WC (− 2.26 cm). Otherwise, a slight increase was observed in HDL-C (+ 0.02 mmol/L). HIIT did not produce any significant changes in TG (− 1.29 mmol/L).

**Conclusions:**

HIIT improves certain clinical aspects in people with MetS (BG, SBP, DBP and WC) compared to people with MetS who do not perform physical exercise. Plausible physiological changes of HIIT interventions might be related with large skeletal muscle mass implication, improvements in the vasomotor control, better baroreflex control, reduction of the total peripheral resistance, increases in excess post-exercise oxygen consumption, and changes in appetite and satiety mechanisms.

## Background

Despite the efforts from national and international bodies to promote healthy behaviours and prevent physical inactivity, global physical inactivity levels have failed to come down over the last decade [1, 2]. In 2016, global physical inactivity prevalence was 27.5%, which means that more than one of every four adults do not meet the minimum recommended physical activity levels [3, 4]. Not meeting the recommended levels of physical activity increases the risk of developing chronic diseases such as obesity, hypertension, type 2 diabetes, osteoporosis, and cancer, and increases the mortality risk [5, 6].

Within these diseases, Metabolic Syndrome (MetS) has one of the highest mortality rates [7]. MetS is defined as a cluster of cardiovascular risk factors that includes elevated blood glucose (BG), low high-density lipoprotein (HDL-C), high triglycerides (TG) levels, high systolic blood pressure (SBP), high diastolic blood pressure (DBP) and increased waist circumference (WC) [8]. It is further agreed that an individual is diagnosed with MetS when three or more of the aforementioned risk factors are present. Even though MetS is responsible for many deaths, it is a condition that is underdiagnosed and therefore undertreated because it is largely asymptomatic [9]. For example, the reported prevalence of MetS in the United States is 35%, however this figure rises to 50% for adults aged 60 and over [10, 11]. In this regard, MetS is a strong predictor of cardiovascular and all-cause mortality. Thus, it is imperative to diagnose and treat individuals with metabolic syndrome effectively [12–14].

The standard treatment for MetS is to prescribe pharmaceuticals for the treatment of the individual risk factors (i.e. hypertension, diabetes, etc), which also brings potentially adverse side effects, such as gastrointestinal problems, arrhythmias, weight gain, insomnia, dizziness, asthenia, etc. [15, 16]. However, evidence suggests that an appropriate lifestyle can help manage and prevent MetS and its associated factors [17]. More specifically, daily physical activity (PA) has been shown to reduce most of the MetS risk factors, and therefore MetS itself [18, 19]. Thus, exercise prescription should be considered as a non-pharmacological, non-invasive, first-line, low-cost treatment to improve MetS. There is evidence that shows strong links between exercise and reducing the prevalence of MetS [20], likely a result of increased caloric expenditure and structural changes in muscle [21, 22].

Lack of time is often cited as one of the main reasons not to meet the recommended PA levels [23]. Because of the time constraints, there is a growing interest in developing alternative approaches to exercise that require shorter work times and lower training volumes, yet still elicit physiological benefits similar to more traditional exercise bouts.

High Intensity Interval Training (HIIT) on MetS is believed to be one of the most time-efficient training modes that have been recently developed [24]. HIIT consists of bouts of exercise at high intensity interspersed by periods of active/passive recovery. HIIT training combinations, by modifying work and rest ratios, are infinite. With shorter training times, it has been suggested that HIIT training can induce similar benefits as prolonged training on cardiorespiratory fitness and muscle oxidative capacity [25, 26].

Nonetheless the prescription of HIIT to inactive individuals that suffer MetS is not without controversy [27], as HIIT is deemed unsafe by some authors. In the literature, we find that after 24 h after a bout of HIIT in patients with cardiometabolic diseases, the adverse responses (cardiac arrest or myocardial infarction) to HIIT is around 8% (most of them were mild in nature), which is slightly higher than seen after Moderate Intensity Continuous Training (MICT) [28]. Although caution must be taken before high intensity training in people with cardiovascular diseases, recent systematic reviews of randomised controlled trials found that the number of adverse events is low [29, 30] and these percentages are much lower in asymptomatic people [31, 32], so this type of training could classified as safe under supervision. In addition to suggesting that HIIT is potentially less safe than MICT, some authors believe that HIIT will not have a public health impact because it is too demanding, eliciting low enjoyment levels and that current inactive people will not adhere to HIIT on the long term, only active individuals will take up this mode of exercise [27]. On the other hand, those that points out HIIT as a promising opportunity to promote a more active behaviour, say that the traditional forms of exercise, including MICT, have been a failure [27]. In regard to behaviour, several studies have reported that HIIT usually offers more enjoyment and affective responses both during and immediately after exercise [33]. Therefore, HIIT may be an alternative to MICT for inducing positive physiological adaptations [28] and doing so in a more enjoyable way [27].

For these reasons there has been an increase in articles published on the effects HIIT has on populations with various chronic diseases, including MetS. Recent studies seem to find that HIIT elicits big morpho-physiological benefit on MetS treatment. Previous reviews have compared the effects HIIT and MICT have on MetS and in single cardiovascular risk factors, and all are in agreement that HIIT can produce similar health benefits in MetS components compared to MICT, yet in a shorter time frame [34–36]. However, to date, no review or meta-analysis has compared the effects of HIIT to non-exercising controls in MetS variables.

Therefore, the purpose of this meta-analysis is to review the literature of randomised controlled trials (RCT) in regard to the effectiveness of HIIT interventions on MetS variables (BG, HDL-C, TG, SBP, DBP, and WC) in adults compared to non-exercise controls.

## Methods

In order to accomplish our objectives, this review followed the Preferred Reporting Items for Systematic Reviews and Meta-Analyses (PRISMA) guidelines [37]. PRISMA checklist can be found in Appendix 1.

### Study selection and eligibility criteria

To be included in the meta-analysis, each study had to fulfil the following criteria: a) Studies had to be randomised controlled trials (RCT), lasting at least 3 weeks, that analysed the effects of HIIT in at least one variable of MetS (BG, HDL-C, TG, SBP, DBP and/or WC) in people with MetS [8] (Table 1); b) all participants in the studies had to be aged ≥18 years;; c) sample size in the post-test had to be higher than 4 participants per group; d) studies had to include a non-exercising control group; e) studies had to report enough statistical data to calculate the effect sizes; f) studies had to be published before January 2020; and g) studies had to be written in English or Spanish. Animal studies, review articles, acute exercise studies, and nonrandomised-controlled trials were excluded.

**Table 1:** Clinical cut off values of Metabolic Syndrome components

Component	Clinical Cut Off Value
Blood Glucose OR (taking anti diabetic medication)	> 100 mg/dL
High-density lipoprotein OR (taking medication for reduced HDL-C)	< 40 mg/dL in males; < 50 mg/dL in females
Triglycerides	> 150 mg/dL
Systolic Blood Pressure / Diastolic Blood Pressure OR (taking anti-hypertensive medication)	> 130 mmHg / > 85 mmHg
Waist Circumference	> 102 cm in males; > 88 cm in females^a^

^a^It is recommended that the International Diabetes Federation cut points be used for non-Europeans and either the International Diabetes Federation cut points used for people of European origin until more data are available

### Search strategy

Potential studies were identified using a systematic search process. First, the following bibliographical databases were searched: Cochrane Library, Embase, PubMed, Sportdiscus, and Web of Science, with the following search terms included in Boolean search strategies: (metabolic syndrome [tiab] OR metabolic syndrome [mesh] OR comorbidities [tiab] OR comorbidities [mesh] OR cardiometabolic disease [tiab] OR cardiometabolic disease [mesh]) AND (HIIT [tiab] OR HIIT [mesh] OR high intensity interval training [tiab] OR high intensity interval training [mesh] OR interval training [tiab] OR interval training [mesh]).

The search was limited to publication dates (to “December 31^st^, 2019”). The reference lists of the studies recovered were hand searched to identify potentially eligible studies not captured by the electronic searches. Two reviewers (I.S.T. and M.A.C.) independently screened the title, abstract and reference list of each study to locate potentially relevant studies, and once hard copies of the screened documents were obtained. The reviewers also attempted to identify articles that met the selection criteria. A third external reviewer (A.L.V.) was consulted to resolve discrepancies regarding the selection process.

### Data extraction and quality assessment

To guarantee the maximum objectivity possible, a codebook was produced that specified the standards followed in coding each of the characteristics of the studies. The outcome measures were BG (mmol/L), HDL-C (mmol/L), TG (mg/dL). SBP (mmHg), DBP (mmHg), and WC (cm).

A complete assessment of the level of risk of bias of the included studies was made following The Cochrane Collaboration’s tool for assessing the risk of bias in randomised trials [38]. The methodological quality of the studies selected was evaluated using the Physiotherapy Evidence Database Scale (PEDro) [39]. A total score out of 10 is derived for each study, adding the criteria that are achieved, a PEDro score ranging from 6 to 10 is indicative of high quality, 4–5 indicates fair quality, and scores of 3 or less indicate poor quality [40]. To assess the inter-coder reliability of the coding process, two researchers coded all the selected studies, including methodological quality assessment and risk of bias. For the quantitative moderator variables, intra-class correlation coefficients (ICCs) were calculated, whereas for the qualitative moderator variables, Cohen’s kappa coefficients were applied. On average, the ICC was 0.96 (range 0.93–1.0) and the kappa coefficient was 0.98 (range 0.95–1.0), which can be considered highly satisfactory [41]. The inconsistencies between the two coders were resolved by consensus or by consulting with a third reviewer. The datasets used and/or analysed during the current study are available from the corresponding author on reasonable request.

### Statistical analysis

All outcomes were reported as means and standard deviations (SD). The standardized mean differences (SMD) were calculated to determine Cohen’s d for each study. For each of the five outcome measures (BG, HDL-C, TG, SBP, DBP and WC), an effect size was calculated as the average difference between the post-test and pre-test change scores of the experimental and control groups: $D=\left({m}_{Post}^E-{m}_{Pre}^E\right)-\left({m}_{Post}^C-{m}_{Pre}^C\right)$ [42]. Negative D values indicated a better result for the intervention group (INT) than for the control one. Separate meta-analyses were performed for each outcome measure. For each, an average effect size (*D*_+_) and a 95% CI were calculated by assuming a fixed-effects model, with the inverse variance as the weighting factor [43]. Heterogeneity of the effect sizes across studies was assessed by means of Cochrane *Q* statistic and the *I*^*2*^ index. A forest plot was also constructed for each meta-analysis. Lack of homogeneity was considered for Cochrane *Q* tests with *p* < 0.10 and/or for *I*^2^ indices. The forest plots were carried out with the Review Manager (RevMan) software package (version 5.5 for OSX, The Nordic Cochrane Centre, The Cochrane Collaboration, 2014, Copenhagen, Denmark).

## Results

### Study selection

**Fig. 1:**
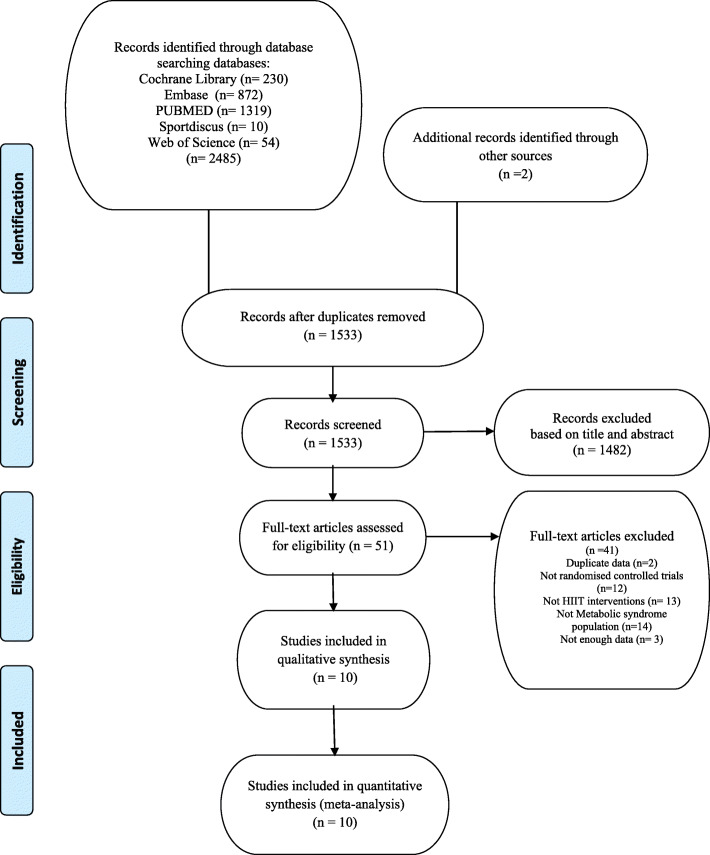
Flow chart of the selection studies in the meta-analysis

Our search strategy resulted in 2487 references. Of these, 954 were removed as duplicates after the first screening, and 1482 references were removed based on the title and abstract. Two other studies had duplicated data, 12 were not randomised controlled trials, 14 studies did not apply HIIT in their interventions, 10 did not include participants with METS, and 3 did not have enough data to calculate effect size. Finally, 10 studies that met the selection criteria were identified [44–53]. Figure 1 shows the flow chart of the selection process of studies.

### Descriptive characteristics of the studies

The main characteristics of each of the studies are presented in Table 2. The studies selected were conducted between 2007 and 2018. Six studies were carried out in Spain, two in Norway, one in Iran, and one in Brazil. The total sample size was 355 in the HIIT intervention groups and 174 in the control groups. Two studies included only men [51], one study included only women [44] and eight trials included men and women [45–50, 52, 53]. The length of the HIIT interventions ranged from 3 to 24 weeks and the weekly training frequency was 3 sessions per week for 9 trials [44–51], 4 sessions per week in one trial [53] and 5 sessions per week in another trial [54]. Type of exercise was cycling in 7 trials [45–49, 51, 53] and walking/running on a treadmill in 3 trials [44, 50, 52].

**Table 2:** Characteristics of the studies included in the meta-analysis

Paper/Country	Participants/ Gender	Duration (Weeks)	Frequency (Days/week)	Exercise training characteristics	Outcome
Alvarez et al. 2018 [44] (Brazil)	HIIT DYSHG: 12 CG: 12 Female	16	3	8 to 14 bouts of 30 to 58 s of jogging/running at 90% HRreserve, interspersed with recovery periods at 70% HRreserve that lasted between 120 s and 96 s. The number of bouts and the duration of each interval increased every week, duration of the recovery periods shortened every week.	HIIT improved BG, HDL-C, TG, BP, TC, LDL-C, endurance performance, body composition and in women in the DYSHG group.
Morales Palomo et al. 2017 [45] (Spain)	HIIT (TRAIN): 23 CG: 26 Mixed	16	3	4 bouts of 4 min of pedalling at 90% of maximal HR interspersed with 3-min active recovery periods at 70% maximal HR.	HIIT reduced BG, SBP, DBP, WC, body weight and BMI. TG levels were not affected by training.
Morales Palomo et al. 2019 [46] (Spain)	HIIT (4HIIT): 32 CG: 22 Mixed	16	3	4 bouts of 4 min of pedalling at 90% of maximal HR interspersed with 3-min active recovery periods at 70% maximal HR.	HIIT significantly reduced body weight, WC and MAP. BG, HDL-C, TG levels were not changed significantly.
Mora Rodriguez et al. 2017 [47] (Spain)	HIIT (TRAIN): 23 CG: 23 Mixed	24	3	4 bouts of 4 min of pedalling at 90% of maximal HR interspersed with 3-min active recovery periods at 70% maximal HR.	HIIT resulted in a significant decrease in WC and mean arterial blood pressure. No significant changes in BG, HDL-C and TG levels in HIIT group.
Mora Rodriguez et al. 2018a [48] (Spain)	HIIT (TRAIN): 18 CG: 16 Mixed	24	3	4 bouts of 4 min of pedalling at 90% of maximal HR interspersed with 3-min active recovery periods at 70% maximal HR.	HIIT resulted in a significant reduction in body weight, percentage of body fat, WC and MAP. HIIT did not elicit changes in TG, BG and HDL-C.
Mora Rodriguez et al. 2018b [49] (Spain)	HIIT (TRAIN): 23 CG: 22 Mixed	16	3	4 bouts of 4 min of pedalling at 90% of maximal HR interspersed with 3-min active recovery periods at 70% maximal HR.	HIIT resulted in significant decrease in SBP, DBP and WC. No significant changes in TG levels in HIIT group.
Mora Rodríguez et al. 2019 [50] (Spain)	HIIT (TRAIN): 76 CG: 20 Mixed	16	3	Twenty minutes continuous at 70% of HRmax followed by 4 bouts of 3 min of walking/running at 90% of HRmax interspersed with a 3-min active recovery at 70% of HRmax between intervals.	HIIT resulted in significant decrease in BG, WC and MAP. No significant changes in TG levels in HIIT group.
Sari-Sarraf et al. 2015 [53] (Iran)	HIIT (HIIT2):11 CG: 11 Male	16	3	HIIT2: 5 bouts of 2 min cycling with 1-min recovery utilizing undulating intensities (80–100% VO_2peak_).	HIIT resulted in significant decrease in, BG, TG, SBP, DBP and WC. No significant changes in HDL-C levels in HIIT group.
Stensvold et al. 2010 [51] (Norway)	HIIT (AIT): 11 CG: 11 Mixed	12	5	4 min intervals of walking/running at 90% of HRmax interspersed with 3 min active recovery periods at 70% of HRmax.	HIIT decreased SBP and DBP.
Tjønna et al. 2008 [52] (Norway)	HIIT (AIT): 11 CG: 11 Mixed	16	4	4 bouts of 4 min of pedalling at 90% of maximal HR interspersed with 3-min active recovery periods at 70% maximal HR.	HIIT resulted in significant decrease in DBP, SBP, and WC. HDL-C significantly increased in HIIT group. No significant changes in TG and BG levels in HIIT group.

*BG* Blood glucose, *BP* Blood Pressure, *BMI* Body Mass Index, *CG* Control group, *DBP* Diastolic blood pressure, *DYSHG* Dyslipidemia and high blood glucose, *HBA1c* Haemoglobin A1c, *HDL-C* High-density lipoprotein, *HR* Heart rate, *HRpeak* Heart rate peak, *HRreserve* Heart rate reserve, *HIIT* High intensity interval training, *LDL-C* Low-density lipoprotein, *MAP* Mean Arterial Pressure, *MICT* Moderate intensity continuous training, *SBP* Systolic blood pressure, *TC* Total Cholesterol, *TG* Triglycerides, *VO*_*2peak*_ Peak oxygen uptake, *WC* Waist circumference

### Quality of the selected studies

The mean score obtained with the PEDro quality scale was 6.7 (minimum score being 5, and the maximum score being 7, higher scores indicating better quality). All of the studies stated the eligibility criteria. Nine studies had the participants randomly allocated to groups, and all had both groups matched at baseline. No studies performed blinding of any kind to the subjects and/or consultants that measure at least one variable. All studies reported results of between-groups statistical analysis and provided point estimates for effect size. PEDro scale of each study can be found in Appendix 2.

### Risk of bias

The risk of bias was assessed with the Cochrane Risk of Bias tool. The risk of bias assessment results can be observed in Appendix 2. According to the Cochrane risk of bias tool, most items (60%) were classified as low risk of bias.

### Effect sizes

**Fig. 2:**
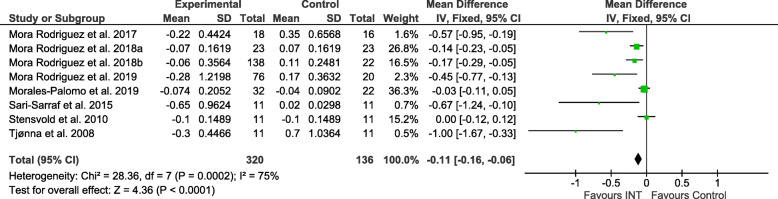
Forest plot of mean difference in blood glucose (BG) of studies included

**Fig. 3:**
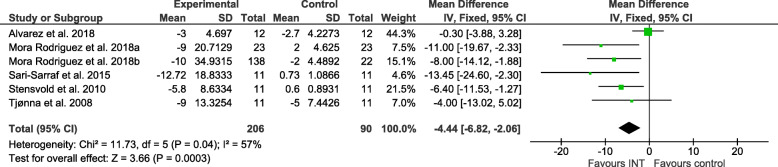
Forest plot of mean difference in systolic blood pressure (SBP) of studies included

**Fig. 4:**
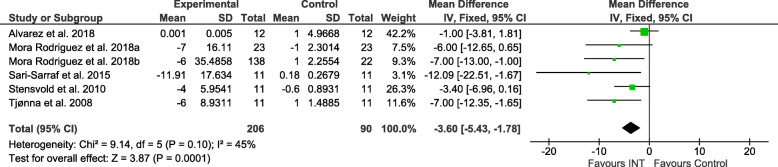
Forest plot of mean difference in diastolic blood pressure (DBP) of studies included

**Fig. 5:**
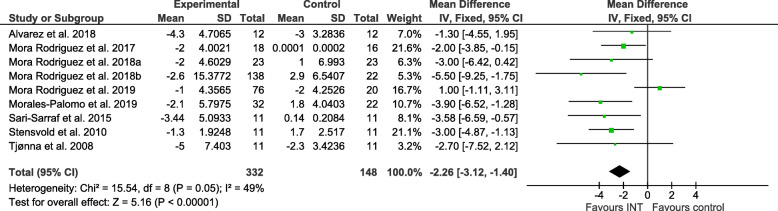
Forest plot of mean difference in waist circumference (WC) of studies included

**Fig. 6:**
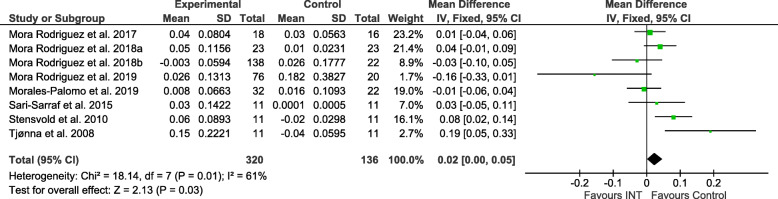
Forest plot of mean difference in high-density lipoprotein (HDL-C) of studies included

**Fig. 7:**
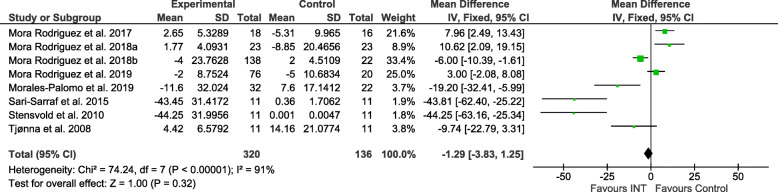
Forest plot of mean difference in triglycerides (TG) of studies included.

Figures 2, 3, 4, 5, 6 and 7 show the main results and forest plots for each of the meta-analyses. Compared with control groups, HIIT groups showed significant reductions in **BG** [*D*_+_ = − 0.11 mmol/L (95% CI: − 0.16 to − 0.06); *p* < 0.0001; SMD: -0.56 (95% CI: − 0.77 to − 0.34). Fig. 2.], **SBP** [*D*_+_ = − 4.44 mmHg (95% CI: − 6.82 to − 2.06); *p* = 0.0003; SMD: -0.48 (95% CI:-0.75 to − 0.20). Fig. 3.], **DBP** [*D*_+_ = − 3.60 mmHg (95% CI: − 5.43 to − 1.78); *p* = 0.0001; SMD: -0.49 (95% CI:-0.76 to − 0.21). Fig. 4.], and **WC** [*D*_+_ = − 2.26 cm (95% CI: − 3.12 to − 1.46); *p* < 0.00001; SMD: -0.44 (95% CI:-0.65 to − 0.23). Fig. 5.] However, a significant increase was observed between groups for **HDL-C** [*D*_+_ = 0.02 mmol/L (95% CI: 0.00 to 0.02); *p* = 0.03; SMD: 0.00 (95% CI:-0.22 to 0.21). Fig. 6]. No differences were found in **TG** [*D*_+_ = − 1.29 mg/dL (95% CI:: − 3.83 to 1.25); *p* = 0.32; SMD: -0.13 (95% CI:-0.35 to 0.09). Fig. 7].

## Discussion

The primary finding of this meta-analysis was that HIIT improved BG, SBP, DBP and WC in individuals with MetS, however HDL-C increase slightly and it did not have any effect on TG.

The reduction observed in BG after HIIT might be explained by increases in skeletal muscle mass, blood flow, and insulin receptors, along with increased disposal of glucose in the skeletal muscle, all as a function of the physical exercise [54]. In addition, there is a known increase in skeletal muscle GLUT-4 expression elicited by training [55] which may be greater following HIIT compared to MICT [56, 57]. HIIT also results in greater recruitment of type II skeletal muscle fibres [58], which may explain the fact that exercise intensity correlates positively with insulin sensitivity [59], an indication that HIIT could potentially be more effective than MICT in managing BG. In the long term, regular exercise, given the acute inflammatory response to exercise partly mediated by IL-6 may protect from low-grade inflammation and thus against insulin resistance [60]. Observed effects of aerobic exercise programs on glucose are similar to those reported in HIIT interventions [61].

The HIIT reduction of SBP and DBP (~ 4 mmHg) has an important clinical impact, since decreases of as little as 2 mmHg reduce the risk of developing coronary artery diseases, myocardial infarction, stroke, and mortality incidence [62–64]. Mechanisms for these reductions in blood pressure are not entirely clear, although enhanced baroreflex control of the sympathetic nerve activity, reduced circulation of catecholamines (norepinephrine), reduced total peripheral resistance, and changes in vasodilator and vasoconstrictor factors are plausible explanations, as these are all elicited by exercise [65–67]. While the role intensity plays on reducing SBP and DBP is not fully understood, compared to other modes of training, HIIT appears to be more potent in reducing SBP and DBP [68, 69].

Lastly, HIIT resulted in a significant reduction in WC of − 2.26 cm. It has been previously stated that a reduction of 4 cm might be clinically relevant [70]. Intraabdominal adipose tissue is a major contributor to MetS [71]. Despite not knowing the precise mechanisms, it has been suggested that the WC reductions might be linked to changes in excess post-exercise oxygen consumption (EPOC), greater fat oxidation, and changes in appetite and satiety mechanisms [72]. EPOC is positively correlated with exercise intensity, thus HIIT has a more powerful effect on EPOC than MICT [73]. Gaitanos et al. [74] suggests that HIIT results in greater fatty acids transport because during the latter stages of HIIT sessions anaerobic glycogenolysis is inhibited, resulting in ATP having to be resynthesised mainly from PCr degradation and Triglycerol stores. HIIT has also been reported to improve appetite control by reducing average TNF-Alpha, PYY, and Ghrelin concentrations, and increasing GLP-1 [75]. Another meta-analysis [76] reported a 3 cm reduction in WC induced by MICT in overweight and obese individuals, showing similar decreases in comparison with the HIIT group in that particular meta-analysis and slightly higher than in our study. Nevertheless, the effect of strength training alone on WC has shown to be less potent (− 1.4 cm) [52].

The present meta-analysis showed a slight increase in HDL-C and it did not find any changes in TG, which agrees with a previous meta-analysis [61]. Compared to MICT, HIIT is not superior in altering blood lipids in adults [77], however the mechanisms behind this are not clear and should be addressed in future studies.

The MetS variables that appear to be most sensitive to HIIT are SBP and DBP, thus suggesting that HIIT as a possible first-line treatment for BP. The other MetS variables improved by HIIT improved by the same magnitude as seen with MICT. However, the nature of HIIT is that sessions require less time and often offer greater enjoyment and may therefore be more efficient than MICT.

Despite the mounting evidence that favours using HIIT to improve indices of cardiometabolic health, the potential impact on public health is still subject to debate regarding its safety and adequacy in terms of enjoyment and adherence [27]. Some research in the past has claimed that there is a negative relationship between exercise intensity and affect, thus suggesting this could be detrimental for exercise adherence [78, 79]. However, a recent meta-analysis and systematic review reported that positive affective responses may be obtained from HIIT [33], the rest intervals in HIIT may be responsible for this response by helping reduce discomfort [80]. Recent data show that in healthy non-obese individuals, HIIT is more enjoyable than prolonged continuous exercise due to it being more time-efficient and because of the repeated stimulus changes [81]. On overweight individuals, HIIT is as enjoyable as MICT and with high adherence rates while being performed unsupervised [82]. Also, HIIT appears to become more enjoyable as the training advances, while MICT enjoyment levels remain unchanged as the weeks progress [83], suggesting that in the long term, HIIT appears to be a more suitable mode of exercise. In this meta-analysis there was no drop out difference between control and intervention groups. Data regarding safety of HIIT against MICT shows there are slightly more adverse events following HIIT sessions than MICT sessions [28]. However, a small sample size was employed, thus further research is needed to assess safety of HIIT. Future research should, therefore, focus on optimal training thresholds for HIIT that are effective, enjoyable, and feasible outside to ensure the greatest public health impact.

### Limitations

There are a number of limitations that may affect this meta-analysis that should be taken into consideration. First of all, the sample size of each included study was small, and some scientific criteria were not indicated in some studies, such as lack of follow-up and control of the group activities in the non-exercise groups. Other limitations were that studies employed heterogeneous HIIT protocols, with different duration of bouts and different modes of exercise.

## Conclusions

HIIT interventions showed significant physiological benefits for BG, SBP, DBP and WC reductions, potentially linked with changes in skeletal muscle’s oxidative capacity, arterial peripheral resistance, and EPOC. However, no significant differences were observed between experimental and control groups for TG and HDL-C increased in a non-relevant way. Therefore, HIIT has the potential to have a public health impact on critical components of the metabolic syndrome. In addition, the reduced time commitment of HIIT and equal if not better levels of enjoyment may make HIIT even better than MICT on an individual and population level. There is, however, still a need for additional research to determine the causal mechanism producing the beneficial metabolic changes, and also to assess minimum HIIT thresholds in regard to frequency, rest intervals, etc., to produce optimal outcomes.

## Data Availability

The datasets used and analysed during the current study are available from the corresponding author on reasonable request.

## Appendix 1

Analysis of the selected studies’ methodological quality (*n* = 10).

**Table:**

Study	1	2	3	4	5	6	7	8	9	10	11	Score
Alvarez et al. 2018 [44]	+	–	–	+	–	–	–	+	+	+	+	5
Morales-Palomo et al. 2017 [45]	+	+	+	+	–	–	–	+	+	+	+	7
Morales-Palomo et al. 2019 [46]	+	+	+	+	–	–	–	+	+	+	+	7
Mora Rodriguez et al. 2017 [47]	+	+	+	+	–	–	–	+	+	+	+	7
Mora Rodriguez et al. 2018a [48]	+	+	+	+	–	–	–	+	+	+	+	7
Mora Rodriguez et al. 2018b [49]	+	+	+	+	–	–	–	+	+	+	+	7
Mora Rodriguez et al. 2019 [50]	+	+	+	+	–	–	–	+	+	+	+	7
Sari-Sarraf et al. 2015 [53]	+	+	+	+	–	–	–	+	+	+	+	7
Stensvold et al. 2010 [51]	+	+	+	+	–	–	–	+	+	+	+	7
Tjønna et al. 2008 [52]	+	+	–	+	–	–	–	+	+	+	+	6

The numbers of the columns corresponded to the following items of the PEDro scale:

1. Eligibility criteria were specified (not included in score).
2. Subjects were randomly allocated to groups.
3. Allocation was concealed.
4. The groups were similar at baseline regarding the most important prognostic indicator.
5. There was blinding of all subjects.
6. There was blinding of all therapists who administered the therapy.
7. There was blinding of all consultants who measured at least one key outcome.
8. Measures of at least one key outcome were obtained from more than 85% of the subjects initially allocated to groups.
9. All subjects for whom outcome measures were available received the treatment or control condition as allocated or, where this was not the case, data for at least one key outcome was analysed by intention to treat.
10. The results of between-group statistical comparisons are reported for at least one key outcome.
11. The study provides both point measures and measures of variability for at least one key outcome.

## Appendix 2

Cochrane risk of bias tool of the selected studies (*n* = 10).

**Table:**

Study	1	2	3	4	5	6
Alvarez et al. 2018 [44]	+	–	–	–	+	+
Morales-Palomo et al. 2017 [45]	+	+	–	–	+	+
Morales-Palomo et al. 2019 [46]	+	+	–	–	+	+
Mora Rodriguez et al. 2017 [47]	+	+	–	–	+	+
Mora Rodriguez et al. 2018a [48]	+	+	–	–	+	+
Mora Rodriguez et al. 2018b [49]	+	+	–	–	+	+
Mora Rodriguez et al. 2019 [50]	+	+	–	–	+	+
Sari-Sarraf et al. 2015 [53]	+	+	–	–	+	+
Stensvold et al. 2010 [51]	+	+	–	–	+	+
Tjønna et al. 2008 [52]	+	–	–	–	+	+

Risk of bias assessment of the included studies. (+) indicates low risk of bias, (?) indicates unclear risk of bias, (−) indicates high risk of bias:

1. Random sequence generation (Selection bias).
2. Allocation concealment (Selection bias).
3. Blinding (participants and personnel) (Performance bias).
4. Blinding (outcome assessment) (Detection bias).
5. Incomplete outcome data (Attrition bias).
6. Selective reporting (Reporting bias).

## Abbreviations

BG: Blood Glucose
DBP: Diastolic Blood Pressure
HDL-C: High Density Lipoprotein
HIIT: High Intensity Interval Training
MetS: Metabolic Syndrome
MICT: Moderate Intensity Continuous Training
PEDro: Physiotherapy Evidence Database Scale
RCT: Randomised controlled trial
Revman: Review Manager
SBP: Systolic Blood Pressure
TG: Triglycerides
WC: Waist Circumference
